# Prolonged gabapentin analgesia in an experimental mouse model of fibromyalgia

**DOI:** 10.1186/1744-8069-4-52

**Published:** 2008-11-06

**Authors:** Michiko Nishiyori, Hiroshi Ueda

**Affiliations:** 1 Division of Molecular Pharmacology and Neuroscience, Nagasaki University Graduate School of Biomedical Sciences, 1-14 Bunkyo-machi, Nagasaki 852-8521, Japan

## Abstract

In a new mouse model for generalized pain syndrome, including fibromyalgia, which used intermittent cold stress (ICS), bilateral allodynia in the hindpaw was observed that lasted more than 12 days; thermal hyperalgesia lasted 15 days. During constant cold stress (CCS), mice showed only a transient allodynia. A female prevalence in ICS-induced allodynia was observed in gonadectomized but not in gonad intact mice. Systemic gabapentin showed complete anti-allodynic effects in the ICS model at the one-tenth dose for injury-induced neuropathic pain model, and central gabapentin showed long-lasting analgesia for 4 days in ICS, but not the injury model. These results suggest that the ICS model is useful for the study of generalized pain syndrome.

## Findings

Fibromyalgia syndrome (FMS) is a highly prevalent (~2% of all citizens) chronic pain disease, which has unique characteristics including generalized or widespread allodynia and a female preponderance [1-4]. According to recent reports, physical and psychological stressors are believed to trigger FMS [5-9]. Although there are several trials underway to make existing medicines available for FMS treatment, attempts to develop a compound specific for FMS are yet to be performed. One reason is the lack of established experimental animal models for FMS, although several models have been proposed [10-13]. Here, we report a pharmacological study using a new generalized chronic pain or FMS model, using intermittent cold stress.

We improved the mouse model for dysautonomia, also referred to as the specific alternation of rhythm in temperature (SART) model [14,15]. To develop the stress model of mice with reproducible and long-lasting allodynia and hyperalgesia, we evaluated several different stress paradigms. With regard to a temperature of the cold room, the temperature of 4 ± 2°C gave rise to long-lasting allodynia. At the temperature over 6°C, long-lasting and stable allodynia was not observed. For quick changes in temperature between 4 and 24°C, we used a meshed stainless steel floor in each cage, and transferred mice individually, leaving the stainless floor in the cold room. Trials with different numbers of mice in the same cage, ranging from one to eight mice, revealed that the number of mice in the same cage should be two. Although frequent temperature change is expected to be more stressful, an interval of more than 30 min was estimated to be minimal for the switch between 4 and 24°C. To maintain healthy conditions in the cold room, food pellets and gelatin as the water supply were placed on the floor of the cage in the cold room, owing to the lowered spontaneous activities of mice in such conditions. For stable nociception, an adaptation period of at least 1 h before the nociception tests was necessary.

**Figure 1:**
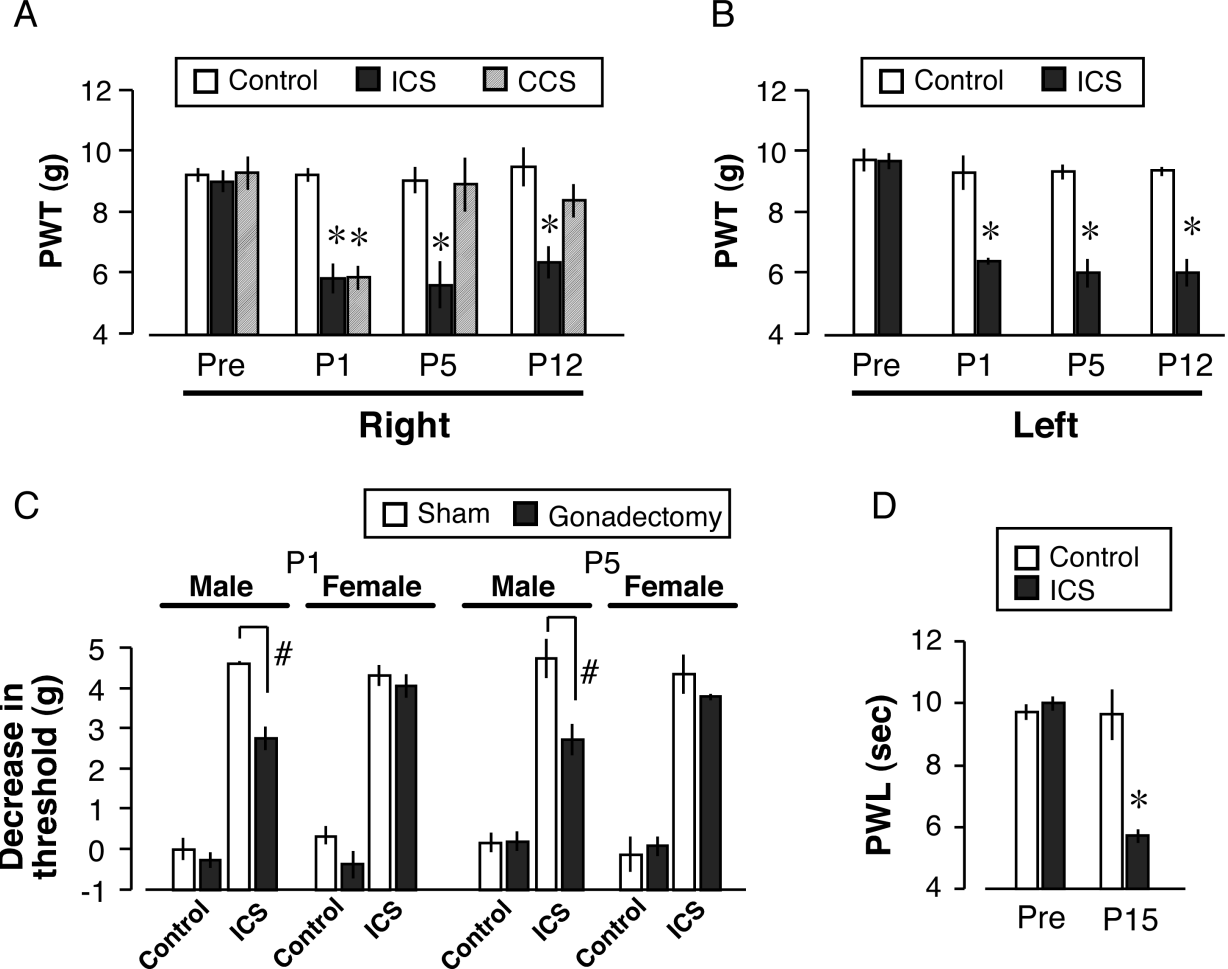
**Long-lasting and bilateral allodynia and hyperalgesia induced by ICS**. A, B: ICS-induced mechanical allodynia on the (A) right and (B) left hind paws. The paw pressure test, using digital von Frey apparatus, was performed at pre-stress (day 0) and at P1, 5 and 12 (days 3, 7 and 14) after ICS/CCS. C: Effects of gonadectomy on ICS-induced mechanical allodynia in male and female mice. Data represent the decrease in the threshold obtained by subtracting the threshold at P1 or P5 from the pre-stress value. D: ICS-induced thermal hyperalgesia. Data represent the means ± S.E.M. from 4–6 individual mice per group. *p < 0.05 versus control group. #p < 0.05 versus sham group.

In the paw pressure test using the digital von Frey apparatus, the control pre-stress threshold of the right paw was 9.5 ± 0.2 g (n = 6), as shown in Fig. 1A. On the other hand, the threshold at day 3 (P1) was significantly decreased to 5.4 ± 0.2 g. Similar thresholds were also found for the left paw, and decreased thresholds on both sides lasted until at least P12 (Figs. 1A, 1B). When CCS was performed, the threshold at P1 was significantly lower (6.1 ± 0.4 g, n = 5) than in the control mice, but no significant change was observed at P5 or P12 (Fig. 1A).

Gonadectomy had no significant influence on the gross behavioral activities. Furthermore, there was no significant change in the control nociceptive threshold in the paw pressure test at 3 weeks after gonadectomy (male: 9.8 ± 0.3 g, female: 9.6 ± 0.3 g). To determine the effects of gonadectomy (or sham operation) on ICS-induced mechanical allodynia, the changes in threshold were determined by subtracting the post-ICS level from the control level (decrease in threshold). As shown in Fig. 1C, the ICS-induced decrease in threshold was 4.5 ± 0.1 g and 4.2 ± 0.2 g for males and females, respectively. There was no apparent gender difference in ICS-induced allodynia. However, the decrease in threshold after ICS was significantly attenuated by gonadectomy of male mice, but not female mice, at P1. Similar results were also observed at P5. In the thermal paw withdrawal test, the control threshold was 10.0 ± 0.2 sec in the thermal paw withdrawal test. After ICS, the threshold was significantly decreased to 5.8 ± 0.1 sec at P15. The degree of hyperalgesia was comparable to that seen in partial sciatic nerve injury [16].

**Figure 2:**
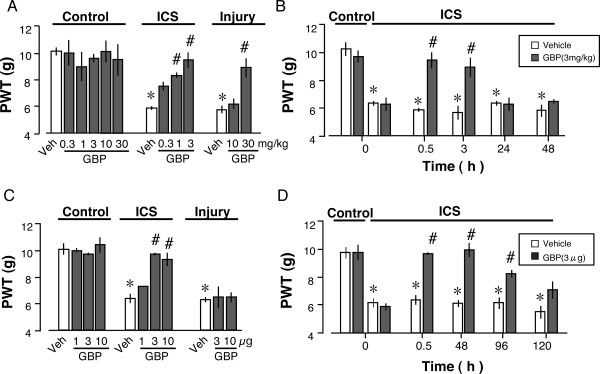
**Analgesic effects of gabapentin in the ICS and neuropathic pain models**. A: Dose-dependent analgesia by gabapentin (i.p.). The paw pressure test was performed at 0.5 h after gabapentin injection at day 7 after ICS or nerve injury. B: Time course of gabapentin (3 mg/kg, i.p.) analgesia in the ICS model. C: Dose-dependent analgesia by gabapentin (i.c.v.). D: Time course of gabapentin (3 μg/kg, i.c.v.) analgesia in the ICS model. Data were normalized to the level in the control plus vehicle group. Data represent the means ± S.E.M. from 4–9 individual mice per group. *p < 0.05 versus control group. #p < 0.05 versus vehicle group.

As shown in Fig. 2A, systemic administration of gabapentin after ICS-treatments dose-dependently reversed the ICS-decreased threshold at 0.5 h in the range of 0.3–3 mg/kg (i.p.). Gabapentin showed significant analgesic effects at 1 and 3 mg/kg in the ICS-model, and at 30 mg/kg in the injury-induced neuropathic pain model. Significant analgesia induced by 3 mg/kg gabapentin was observed at 0.5 and 3 h, but not at 24 or 48 h in the ICS model (Fig. 2B). When gabapentin was administered i.c.v., complete reversal of the ICS-decreased threshold at 0.5 h was observed with 3 or 10 μg gabapentin, which was given at P5 (Fig. 2C). However, no significant analgesia was observed with 3 and 10 μg gabapentin in the injury model. Over the time course of study of gabapentin (i.c.v.) effects, complete and significant reversal of ICS-decreased threshold was also observed at 48 and 96 h, respectively (Fig. 2D). There was some, but not significant, analgesia at 120 h.

In the present study, we demonstrated that intermittent cold stress (ICS) causes abnormal pain, including mechanical allodynia and hyperalgesia, which lasts for more than 2 weeks. Although similar allodynia was also observed at P1 after the constant or CCS, the threshold was reversed to the normal level at P5. Thus, this long-lasting allodynia was specific for intermittent changes in temperature. During the establishment of this model, we examined several paradigms of changes in cold stress. From these attempts, we found that a low temperature for the cold stress and a high frequency of temperature alterations produced a long-lasting and stable allodynia. Based on these results, we determined the optimum paradigm to generate the generalized pain, producing a FMS model in the mouse. This model has several advantages, as no specific apparatus, except for a conventional refrigerator for medical use, is necessary, and the short period of stress application (i.e., three nights) is sufficient to cause long-lasting mechanical allodynia and thermal hyperalgesia.

There are several stress-induced generalized pain syndromes, such as FMS, chronic fatigue syndrome, temporomandibular jaw disorder and irritable bowel syndrome [17]. These pain-related syndromes share a common feature with a female prevalence. In the present study, when the male and female mice were gonadectomized, only male mice showed reversal of ICS-induced allodynia. There was no significant change in the threshold in female mice. As gender hormones are still expected to exist in the adrenal cortex, the endocrine homeostasis may compensate for this endocrine disturbance, but it is not necessarily correct that androgens may play roles in abnormal pain caused by ICS stress. However, as the castration itself did not affect the nociceptive threshold, some contributions of androgens to the abnormal pain are likely to work under the condition of ICS. Therefore, we speculate that this ICS model has a gender hormone-independent female predominance of chronic pain.

Of these pain syndromes described, the intensity of FMS is the highest. In addition to the intensity, FMS is known to have unique widespread allodynia. Indeed, we demonstrated mechanical allodynia on both hind paws and intense thermal hyperalgesia for long periods. All these findings suggest that ICS-stress may be a good model for FMS or related stress-induced pain syndromes, as mentioned above.

The second issue is the potent analgesic actions of gabapentin. Gabapentin is now widely used as a potent analgesic against neuropathic pain. Because of its chemical instability, higher doses are required in clinical use. In the present study, we confirmed that relatively high doses (10–30 mg/kg i.p.) of gabapentin significantly inhibited nerve injury-induced neuropathic pain. Similar effective doses were also observed in chemotherapy (paclitaxel)-induced neuropathic pain in our previous study [18]. In the ICS-model, however, a dose of gabapentin as low as 3 mg/kg (i.p.) was sufficient for the complete inhibition of allodynia/hyperalgesia. It should be noted that central administration of gabapentin also showed potent analgesia, but the analgesia persisted for 4–5 days after a single injection. Gabapentin at 3 μg (i.c.v.) showed complete analgesia in the ICS model, but not at 10 μg in the neuropathic pain model, which is consistent with the previous study that 100 μg of gabapentin is required for the complete analgesia in the neuropathic pain model [19]. Thus the abnormal pain in the ICS model mice provides a better target for gabapentin than that in nerve injury- or paclitaxel-induced neuropathic pain, in which 30 mg/kg of gabapentin is required for complete analgesia [18]. However, it remains to be determined why a single injection of gabapentin has such long-term effects.

Stress-induced pain, as in FMS, is considered to be caused by intense events involving physical and psychological injury and is reinforced by successive stress. In other words, it develops into a vicious cycle of pain-induced pain. Although the site of action for gabapentin analgesia in the ICS model remains to be determined, initial blockade of the pain mechanisms may have relieved the successive cycle. Taking this notion into consideration, the site of action of gabapentin in the ICS model seems to be in the central nervous system. This view seems to be consistent with the finding that a higher dose of central gabapentin was required for nerve injury-induced neuropathic pain than was the case with ICS model. However, this may exclude the possibility that centrally administered gabapentin is quickly adsorbed in the brain tissues and slowly released. According to recent studies, the most likely molecular target is the α 2δ 1 subunit of a voltage-dependent calcium channel [20-22]. Actually, we have demonstrated up-regulation of this subunit expression in the dorsal root ganglion, which may underlie the mechanisms of gabapentin analgesia in the nerve injury- or paclitaxel-induced neuropathic pain model [18]. It is of interest to further examine if the up-regulation of the α 2δ 1 subunit is also involved in the potent gabapentin analgesia in the ICS-model.

In conclusion, the present study demonstrates that the ICS model is a promising candidate for experimental FMS or generalized pain syndrome models. In addition, we found beneficial effects of systemic and central gabapentin in this model in terms of potency and duration.

## Materials and methods

### Animal treatments

Six-week-old male and female C57BL/6J mice weighing 18–22 g were used. These mice were individually kept in a room maintained at 22 ± 2°C, humidity 60 ± 5% and *ad libitum *feeding of a standard laboratory diet and tap water before use. In the intermittent cold stress (ICS) model experiments, two mice per group were kept in a cold room at 4 ± 2°C at 4:30 p.m. on the first day (day 0), with *ad libitum *feeding and agar instead of water. Mice were placed on a stainless steel mesh and covered with plexiglass cage. At 10:00 a.m. the next morning, mice were transferred to the normal temperature room at 24 ± 2°C. After they were placed at the normal temperature for 30 min, mice were put in the cold room again for 30 min. These processes were repeated until 4:30 p.m Mice were then put in the cold room overnight. After the same treatments on the next day, mice were finally taken out from the cold room at 10:00 a.m. on day 3 (post-stress P1) and were kept there for adaptation before nociception tests, which were started at least 1 h later. On the other hand, in the constant or CCS model experiments, mice were kept in the cold room without alternating the environmental temperature for three consecutive nights. The body weight of mice after either ICS or CCS stress, decreased by approximately 10% after 2–3 days, but subsequently recovered to the normal level of unstressed control mice and throughout the experiments. In the neuropathic pain model, nociception tests were carried out 1 week after the partial ligation of sciatic nerve, as previously reported [23]. All experiments were performed in compliance with the relevant laws and institutional guidelines. All procedures were approved by the Nagasaki University Animal Care Committee and complied with the recommendations of the International Association for the Study of Pain (Zimmermann, 1983).

### Gonadectomy

Six-week-old male and female mice were gonadectomized by removing ovaries or testes, respectively under pentobarbital (50 mg/kg i.p.) anesthesia. After the surgery, mice were kept in a soft bed cage with some food inside so that the animals could feed themselves without difficulty in standing. Gonadectomized mice were used for ICS and nociception tests 3 weeks later.

### Nociception tests

In most of the experiments, the paw pressure test was carried out using a digital von Frey apparatus test (Anesthesiometer, IITC Inc., Woodland Hills, USA), as previously reported [16,24]. In this experiment, the threshold (in grams) of given pressure to cause the paw withdrawal behavior of mouse was evaluated. The maximum response threshold was set at 20 g to prevent tissue damage. In some experiments, the thermal paw withdrawal test was carried out using a thermal stimulus (Model 33 Tail Flick analgesia meter, IITC Inc., Woodland Hills, CA, USA), as previously reported [16,24]. A cut-off time was set at 20 seconds to prevent tissue damage. Nociception tests were performed between 12:00 and 4:30 p.m. throughout experiments. The control threshold was determined immediately prior to the start of ICS or CCS (pre-stress). Test thresholds were determined on days 3, 7 and 14, which were represented as post-stress day 1, 5 and 12 (P1, P5 and P12), respectively.

### Drug treatment

Gabapentin, purchased from Sigma-Aldrich (St. Louis, MO), was dissolved in physiological saline or artificial cerebrospinal fluid (aCSF) for intraperitoneal (i.p.) or intracerebroventricular (i.c.v.) treatments, respectively. Gabapentin was given in a volume of 0.1 ml/10 g or 5 μl/mouse for i.p. or i.c.v. injection on day 7 (P5).

### Statistical analysis

All results are expressed as means ± S.E.M. Differences between multiple groups were analyzed using a one-way ANOVA with Scheffe's F multiple comparison post-hoc analysis. Changes in the thresholds were analyzed using an unpaired Student's *t*-test. The criterion of significance was set at *p *< 0.05.

## Competing interests

The authors declare that they have no competing interests.

## Authors' contributions

HU is responsible for experimental design and writing the manuscript. MN is responsible for performance of behavioral analysis. All authors read and approved the final manuscript
